# Changes in the retina and choroid in patients with internal carotid artery stenosis: a systematic review and meta-analysis

**DOI:** 10.3389/fnins.2024.1368957

**Published:** 2024-04-15

**Authors:** Xiaoyu Hou, Chuanhong Jie, Ziqiang Liu, Xuqi Bi, Yu Deng, Yuanyuan Li, Jingying Wang, Weiqiong Zhang

**Affiliations:** Eye Hospital China Academy of Chinese Medical Sciences, Beijing, China

**Keywords:** internal carotid artery stenosis, retina, choroid, optical coherence tomography, optical coherence tomography angiography, meta-analysis

## Abstract

**Background:**

Internal carotid artery stenosis (ICAS) is a prevalent vascular condition associated with ischemic cerebrovascular disease. The ophthalmic artery is the first branch of the internal carotid artery stenosis (ICA). Given the crucial role of the ICA in ocular perfusion, we aimed to assess the thickness and vessel density of the retina and choroid in individuals with ICAS.

**Methods:**

The PubMed and Embase databases were searched from inception to 10 January 2023 for studies evaluating retinal and choroidal changes between ICAS patients and healthy controls using optical coherence tomography (OCT) or optical coherence tomography angiography (OCTA). Data of interest were extracted and analyzed using Stata software version 16.

**Results:**

Thirteen studies involving 419 ICAS eyes and 398 healthy eyes were included. The pooled results demonstrated that the average thickness of peripapillary retinal nerve fiber layer (pRNFL) (WMD = −0.26, 95% CI: −0.45 to −0.08, *P* = 0.005), ganglion cell complex (GCC) (WMD = −0.36, 95% CI: −0.65 to −0.06, *P* = 0.017), and choroid (WMD = −1.06, 95% CI: −1.59 to −0.52, *P* = 0.000), were significantly thinner in patients with ICAS than in healthy controls. The overall vessel density of the radial peripapillary capillaries (RPC) in whole-image scans was lower in ICAS patients than in healthy control subjects (WMD = −0.94, 95% CI: −1.49 to −0.39, *P* = 0.001). No differences were detected in the vessel density of the superficial capillary plexus (SCP) (WMD = −0.84, 95% CI: −1.15 to −0.53, *P* = 0.092), the deep capillary plexus (DCP) (WMD = −0.27, 95% CI: −0.56 to 0.03, *P* = 0.074), or the choriocapillaris (CC) (WMD = −0.39, 95% CI: −1.12 to 0.35, *P* = 0.300).

**Conclusion:**

This systematic review and meta-analysis demonstrated that ICAS can reduce the vessel density of the RPC and the thickness of the retina and choroid. The retinal and choroidal microvasculature is a potential biomarker of the initial signal of ICAS.

**Systematic review registration:**

https://inplasy.com/, identifier NPLASY202410038

## 1 Introduction

Carotid artery stenosis (CAS) is a major risk factor for acute ischemic stroke (AIS). A recent study of approximately 3,500 individuals reported an estimated incidence rate of 4.7% for ipsilateral carotid artery-related AIS over 5 years (Chang et al., 2022). Moreover, ipsilateral carotid stenosis (ICS) has a negative impact on the course of interventional therapy for AIS patients. This may be due to cerebral hypoperfusion and ongoing microembolization of the ischemic region caused by ICS (Viticchi et al., 2023). CAS has become a prominent public health concern. Notably, the ophthalmic artery, which provides blood to ocular tissues, originates from the internal carotid artery stenosis (ICA). Internal carotid artery stenosis (ICAS) usually causes inadequate ocular blood flow, resulting in ischemic ophthalmopathy, such as ocular ischemia syndrome, ischemic optic neuropathy, optic disk, or retinal neovascularization (Kang et al., 2019). Neuro-ophthalmology has attracted increasing interest for the treatment of ischemic ophthalmopathy that originates from ICAS. Ocular symptoms are often the initial signal of carotid artery stenosis, making them a significant factor in warning of ICAS (Durusoy et al., 2022). To prevent impaired visual acuity and reduce stroke risk, it is crucial to promptly identify the correlation between ocular lesions and ICAS.

Optical coherence tomography (OCT) and optical coherence tomography angiography (OCTA) are capable of quantitatively detecting indicators such as choroidal thickness, choroidal vascular index, retinal structure, and retinal vascular density. Therefore, they are currently used as clinical biomarkers for ischemic ophthalmopathy (Phuljhele et al., 2023). As an emerging ophthalmic examination technique, OCT can be used to observe the retinal structure in different layers, and enhanced depth imaging-OCT (EDI-OCT) can improve the visualization of the choroid based on OCT, which is able to quantitively assess the choroidal structure more clearly (Wang et al., 2023). OCTA not only allows qualitative analysis of vascular lesions in various tomographies and regions but also provides quantitative analysis of blood flow density (Xu et al., 2022). Numerous studies have indicated that OCTA can be employed for both the diagnosis and assessment of ophthalmological conditions including diabetic retinopathy, glaucoma, retinal vein occlusion, age-related macular degeneration, and ischemic optic neuropathy, as well as for investigating neurological diseases such as Alzheimer’s and demyelinating disorders (Katsimpris et al., 2022; Nepal et al., 2022; Mohammadi et al., 2023).

Recently, many studies have focused on employing OCT and OCTA to measure and examine the retina and choroid in patients with ICAS (Ala-Kauhaluoma et al., 2021; Li et al., 2021; Kwapong et al., 2022). However, no systematic analysis of OCT and OCTA as complementary diagnostic tools for ICAS is currently available. Therefore, we conducted a systematic review and meta-analysis to compare the morphological structure and vessel density of the retina and choroid in individuals with ICAS to elucidate these findings.

## 2 Materials and methods

This systematic review and meta-analysis was carried out in accordance with both the Preferred Reporting Items for Systematic Reviews and Meta-Analyses (PRISMA) statement and the Meta-analysis of Observational Studies in Epidemiology (MOOSE) guidelines (Stroup et al., 2000; Liberati et al., 2009). Furthermore, the study was registered in the INPLSY platform with the number INPLASY202410038.

### 2.1 Search strategy

From the outset to January 10, 2023, the PubMed and EMBASE databases were searched for studies assessing changes in the association between the retina and choroid in ICAS by using OCT or OCTA. A mixture of key phrases and unbound terms were subsequently used: (“ICAS” OR “carotid artery disease” OR “carotid artery stenosis” OR “ICAS” OR “CAS” OR “carotid stenosis”) AND (“optical coherence tomography” OR “OCT” OR “optical coherence tomography angiography” OR “OCT angiography” OR “OCTA”). To reduce the possibility of overlooking pertinent studies, we additionally conducted a manual search by meticulously examining the references cited in the included studies. The detailed search strategies used are provided in Supplementary Table 1.

### 2.2 Inclusion and exclusion criteria

The inclusion criteria were as follows: (a) written in English; (b) and diagnosis of ICAS determined through Angio-CT or Digital Subtracted Angiography or Ultrasound examinations based on the American Heart Association/American Stroke Association (AHA/ASA) or North American Symptomatic Carotid Endarterectomy Trial (NASCET) (Kirkpatrick, 1998; Meschia et al., 2014). The examination and diagnostic methods were the same for the same patients in the same group; (c) the diagnostic criteria for the degree of ICAS were mild stenosis, with a stenosis rate < 50%; moderate stenosis, with a 50% < stenosis rate < 69%; severe stenosis, with a 70% < stenosis rate < 99%; and carotid occlusion, with a stenosis rate = 100% (Kirkpatrick, 1998); (d) patients with ICAS were included in the observation group, and healthy people were included in the control group; (e) OCT and OCTA measurements are reported as the mean and standard deviation (SD).

The exclusion criteria were as follows: (a) duplicated publications, literature reviews, and other unrelated literature; (b) patients who had ophthalmic diseases that significantly impaired ocular circulation, including glaucoma, nonarteritic anterior ischemic neuropathy, diabetic retinopathy, retinal vein occlusion, and age-related macular degeneration; (c) duplicate study populations; (d) studies with incomplete data.

### 2.3 Data extraction and quality assessment

After removing duplicates, two investigators independently screened the titles, abstracts, and full texts of the studies based on the inclusion and exclusion criteria. Disagreements were resolved through consultation or by discussion with a third reviewer. Data from eligible studies were independently extracted by two reviewers, and any disagreements were resolved through consultation or by discussion with a third reviewer. The following information was extracted: author and publication date, country of publication, type of study, sample size, average age, sex, machine type, thickness of the pRNFL, GCC, macula, and choroid, and vessel densities of the SCP, DCP, RPC, and CC. The quality and risk of bias were assessed by two investigators using the Newtle-Ottawa Scale (NOS) (Stang, 2010). The quality score was determined by three factors: subject selection, comparability, and exposure. A score of 6 or higher was considered to indicate high quality research.

### 2.4 Statistical analysis

The data were analyzed using Stata software version 16. The weighted mean difference (WMD) was used as the measure data effect statistic, and both provided their 95% confidence interval (CI). Cochrane’s Q test and the I^2^ statistic were used to assess the statistical heterogeneity. A random-effects or fixed-effects model was applied for the meta-analysis based on the level of heterogeneity. When I^2^ was ≦ 50%, the fixed effect model was used; when I^2^ was >50%, the random effect model was used (Higgins et al., 2003). A *P*-value < 0.05 was regarded as significant. To prevent differences in results caused by high heterogeneity between studies, we conducted sensitivity analysis and subgroup analysis.

## 3 Results

### 3.1 Literature search

**FIGURE 1:**
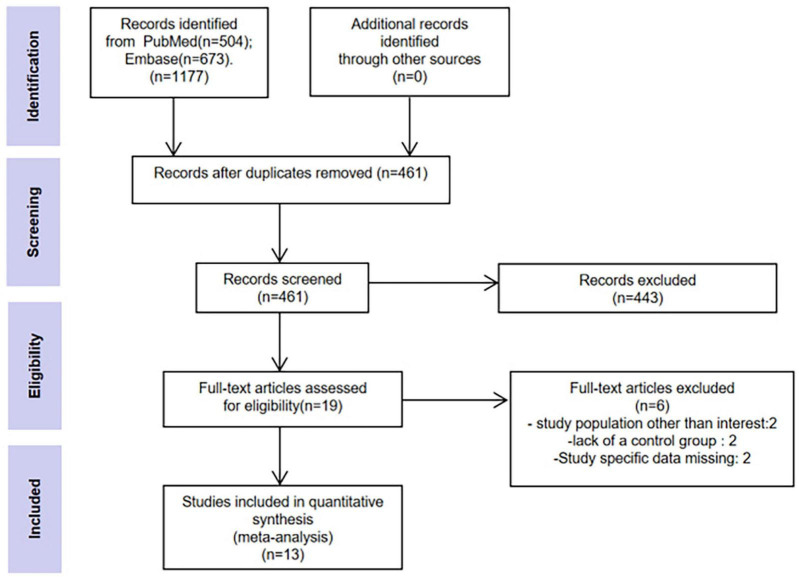
Flow chart of the study selection process.

The initial search identified 1,177 studies that could be relevant, and 716 duplicates were eliminated. After screening the titles and abstracts of the remaining 461 articles, 443 were excluded. After full-text screening, six studies were excluded. Two studies were removed because their population did not meet the interest, two studies lacked a control group, and two studies lacked sufficiently detailed data. Overall, thirteen studies (Heßler et al., 2015; Sayin et al., 2015; Biberoğlu et al., 2017; Çakır et al., 2017; Wang et al., 2017; Lahme et al., 2018; Li et al., 2019; Biberoglu et al., 2020; Dagdelen and Muz, 2021; Pierro et al., 2021; Incekalan et al., 2022; Liu X. et al., 2022; Wan et al., 2022) were included. The screening process is shown in Figure 1.

### 3.2 Characteristics and qualities of the eligible studies

Thirteen studies involving 419 eyes with ICAS and 398 eyes with healthy controls were identified. Eight studies (Heßler et al., 2015; Sayin et al., 2015; Çakır et al., 2017; Lahme et al., 2018; Li et al., 2019; Pierro et al., 2021; Incekalan et al., 2022; Liu X. et al., 2022; Wan et al., 2022) were prospective, while four (Biberoğlu et al., 2017; Wang et al., 2017; Biberoglu et al., 2020; Dagdelen and Muz, 2021) were retrospective in design. The mean age of patients with ICAS was between 62 and 68 years. The majority of the eligible studies recruited patients with moderate to severe ICAS. Of the thirteen studies examined, eleven (Heßler et al., 2015; Sayin et al., 2015; Biberoğlu et al., 2017; Wang et al., 2017; Çakır et al., 2017; Li et al., 2019; Biberoglu et al., 2020; Dagdelen and Muz, 2021; Pierro et al., 2021; Liu X. et al., 2022; Wan et al., 2022) utilized OCT to measure the thickness of the retina and choroid, while five (Lahme et al., 2018; Pierro et al., 2021; Incekalan et al., 2022; Liu X. et al., 2022; Wan et al., 2022) opted for OCTA to evaluate vessel densities of the SCP, DCP, RPC, and CC. We deemed research scoring 6 or above on the NOS scale to be high quality. All of the included studies were high quality studies. Six articles (Heßler et al., 2015; Sayin et al., 2015; Biberoğlu et al., 2017; Incekalan et al., 2022; Liu X. et al., 2022; Wan et al., 2022) scored an NOS of 7, and seven articles (Wang et al., 2017; Çakır et al., 2017; Lahme et al., 2018; Li et al., 2019; Biberoglu et al., 2020; Dagdelen and Muz, 2021; Pierro et al., 2021) scored an NOS of 8. The detailed characteristics of the studies and the NOS scores are listed in Table 1. The quality assessment of the literature is shown in Supplementary Table 2.

**TABLE 1:** Main characteristics of eligible studies.

References	Country	Study design	No. of eyes		Age (year)		M/F		OCT/ OCTA device	OCT type	OCTA-Scan size (mm2)	Outcome variables	Stenosis (%)	NOS score
			**ICAS**	**HC**	**ICAS**	**HC**	**ICAS**	**HC**						
Sayin et al., 2015	Turkey	Prospective	25	25	66.5	65.5	8/17	6/19	Zeiss	SD/EDI-OCT	–	CMT, pRNFL, GCC, CT	≥70	7
Heßler et al., 2015	Germany	Prospective	15	15	62.3	66.4	9/6	7/8	Heidelberg	SD-OCT	–	pRNFL	≥50	7
Çakır et al., 2017	Turkey	Prospective	23	24	67.5	61.4	15/8	12/12	Toronto	SD-OCT	–	CMT,pRNFL	≥50	8
Biberoğlu et al., 2017	Turkey	Retrospective	15	18	64.2	63.7	15/0	18/0	Optovue	SD-OCT	–	pRNFL	≥70	7
Wang et al., 2017	China	Retrospective	49	43	62.0	59.2	15/34	14/29	Heidelberg	EDI-OCT	–	CT	≥70	8
Lahme et al., 2018	Germany	Prospective	25	25	64.5	64.7	–	–	Optovue	–	M3,O4.5	SCP-VD, DCP-VD, RPC-VD	≥70	8
Li et al., 2019	China	Retrospective	61	20	65.1	64.4	42/19	12/8	Heidelberg	EDI-OCT	–	CT	≥50	8
Biberoglu et al., 2020	Turkey	Retrospective	15	18	63.6	63.7	15/0	18/0	Optovue	SD-OCT	–	CT	≥70	8
Pierro et al., 2021	Italy	Prospective	30	30	68.3	65.2	17/13	16/14	Topcon	SD-OCT	M3,O4.5	pRNFL,GCC,CT, SCP-VD, DCP-VD, RPC-VD, CC-VD	≥50	8
Dagdelen and Muz, 2021	Turkey	Retrospective	31	53	63.5	58.5	18/13	28/25	Heidelberg	SD-OCT	–	CMT,pRNFL	≥50	8
Liu X. et al., 2022	China	Prospective	34	40	64.6	62.0	27/7	26/14	Zeiss	EDI-OCT	M3,O6	CMT,pRNFL,GCC, CT, SCP-VD, DCP-VD, RPC-VD	≥50	7
Wan et al., 2022	China	Prospective	53	40	64.1	63.7	6/47	5/35	VG 200	SS-OCT	–	CT, CC	≥50	7
Incekalan et al., 2022	Turkey	Prospective	43	47	65.2	64.1	30/13	33/14	Optovue	–	M3,O4.5	RPC-VD, pRNFL,CT	≥50	7

ICAS, internal carotid artery stenosis; HC, healthy controls; M/F, male/female; OCT, optical coherence tomography; OCTA, optical coherence tomography angiography; SD-OCT, spectral-domain OCT; EDI-OCT, enhanced depth imaging OCT; SS-OCT, swept-source OCT; M, macular scan; O, optic nerve head scan; CMT, central macular thickness; pRNFL, peripapillary retinal nerve fiber layer; GCC, ganglion cell complex; CT, choroidal thickness; VD, vessel density; SCP, superficial capillary plexus; DCP, deep capillary plexus; CC, choriocapillaris; RPC, radial peripapillary capillaries plexus; NOS, Newcastle-Ottawa Scale.

### 3.3 Meta-analysis

All pooled estimates are summarized in Table 2. The detailed forest plots (ICAS vs. HC) are shown in Supplementary Figure 1.

**TABLE 2:** Differences in OCT/OCTA measurements between ICAS and healthy controls.

Outcome indicators	No. of studies	No. of eyes		Heterogeneity		WMD (95% CI)	Overall effect		Egger’s test
		**ICAS**	**HC**	**I^2^ (%)**	***P***		***Z***	***P***	
**pRNFL**									
Average	8	216	252	4.8	0.393	−0.261 [−0.45, −0.08]	−2.783	0.005	0.963
Inferior	4	105	133	0	0.628	−0.19 [−0.45, 0.06]	−1.477	0.140	0.405
Nasal	4	105	133	67.2	0.027	0.03 [−0.44, 0.49]	0.110	0.912	0.888
Superior	4	105	133	0	0.491	−0.10 [−0.36, 0.16]	−0.750	0.453	0.383
Temporal	4	105	133	0	0.530	0.00 [−0.26, 0.26]	0.012	0.990	0.691
CMT	4	113	142	66.2	0.031	−0.36 [−0.80, 0.08]	−1.596	0.110	0.733
GCC	3	89	95	34.3	0.218	−0.36 [−0.65, −0.06]	−2.392	0.017	0.477
CT	8	310	263	87.9	0.000	−1.06 [−1.59, −0.52]	−3.891	0.000	0.538
SCP-VD	3	87	95	90.7	0.000	−0.84 [−1.15, −0.53]	−1.684	0.092	0.576
DCP-VD	3	87	95	0	0.486	−0.27 [−0.56, 0.03]	−1.786	0.074	0.805
RPC-VD	4	132	142	78.3	0.003	−0.94 [−1.49, −0.39]	−3.350	0.001	0.215
CC-VD	2	83	70	79.8	0.026	−0.39 [−1.12, 0.35]	−1.030	0.300	–

#### 3.3.1 Thickness of the retina and choroid between ICAS patients and healthy controls

Eight studies (Heßler et al., 2015; Sayin et al., 2015; Biberoğlu et al., 2017; Çakır et al., 2017; Dagdelen and Muz, 2021; Pierro et al., 2021; Incekalan et al., 2022; Liu X. et al., 2022) compared the average thickness of the pRNFL between ICAS patients and healthy controls. The average pRNFL thickness in ICAS patients was significantly lower than that in healthy controls (WMD = −0.26, 95% CI: −0.45 to −0.08, *P* = 0.005), and no heterogeneity was detected among these studies (I^2^= 4.8%, *P* = 0.393). Subsequently, the pRNFL thickness in different quadrants was compared in Four studies (Heßler et al., 2015; Sayin et al., 2015; Dagdelen and Muz, 2021; Liu X. et al., 2022). No significant differences were found between ICAS patients and healthy controls in the four quadrants (inferior: WMD = −0.19, 95% CI: −0.45 to 0.06, *P* = 0.140; nasal: WMD = 0.03, 95% CI: −0.44 to 0.49, *P* = 0.912; superior: WMD = −0.10, 95% CI: −0.36 to 0.16, *P* = 0.453; temporal: WMD = 0.00, 95% CI: −0.26 to 0.26, *P* = 0.990). Four studies (Sayin et al., 2015; Çakır et al., 2017; Dagdelen and Muz, 2021; Liu X. et al., 2022) compared macular thickness between ICAS patients and healthy controls and showed no significant differences in thickness (WMD = −0.36, 95% CI: −0.80 to 0.08, *P* = 0.110). Three studies (Sayin et al., 2015;18, Pierro et al., 2021; Liu X. et al., 2022) compared the thickness of the GCC between ICAS patients and healthy controls. The GCC thickness was lower in ICAS patients than in healthy controls, with a weighted mean difference (WMD) of −0.36 (95% CI: −0.65 to −0.06, *P* = 0.017). There was no heterogeneity among these studies (I^2^= 34.3%, *P* = 0.218). Eight studies (Sayin et al., 2015; Wang et al., 2017; Li et al., 2019; Biberoglu et al., 2020; Pierro et al., 2021; Incekalan et al., 2022; Liu X. et al., 2022; Wan et al., 2022) compared the thickness of the choroid between ICAS patients and healthy controls. The choroidal thickness in ICAS patients was significantly lower than that in healthy controls (WMD = −1.06, 95% CI: −1.59 to −0.52, *P* < 0.00001).

#### 3.3.2 The vessel density of the retina and choroid between ICAS patients and healthy controls

Three studies (Lahme et al., 2018; Pierro et al., 2021; Liu X. et al., 2022) compared the vessel density of the SCP and DCP between ICAS patients and healthy controls. No significant difference was observed in the vessel density of the SCP (WMD = −0.84, 95% CI: −1.15 to −0.53, *P* = 0.092) or DCP (WMD = −0.27, 95% CI: −0.56 to 0.03, *P* = 0.074) between ICAS patients and healthy controls. Four studies (Lahme et al., 2018; Pierro et al., 2021; Incekalan et al., 2022; Liu X. et al., 2022) compared the vessel density of RPC in ICAS patients and healthy controls. The vessel density of the RPC in ICAS patients was significantly lower than that in healthy controls (WMD = −0.94, 95% CI: −1.49 to −0.39, *P* = 0.001). Two studies(Pierro et al., 2021; Wan et al., 2022)compared the vessel density of the choriocapillaris between ICAS patients and healthy controls. There was no significant difference in the vessel density of the choriocapillaris (WMD = −0.39, 95% CI: −1.12 to 0.35, *P* = 0.300) between ICAS patients and healthy controls.

#### 3.3.3 Sensitivity analysis

The reliability of the outcomes of this study was confirmed through a sensitivity analysis of the outcome indicators, with each study removed one by one. The results indicated that the combined values of the various study effects remained stable (Supplementary Figure 2).

#### 3.3.4 Subgroup analysis

Subgroup analysis was conducted on relevant ICAS patient indicators based on the degree of carotid artery stenosis and type of OCT equipment. The results demonstrated no significant change in combined effect outcomes (Supplementary Table 3).

#### 3.3.5 Publication bias

**FIGURE 2:**
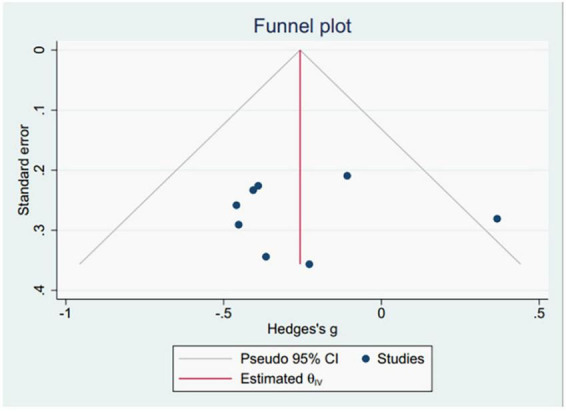
The funnel plots of pRNFL thickness.

This study utilized the average pRNFL thickness as the primary indicator for drawing funnel plots. The results demonstrated that the funnel plot distribution was not entirely symmetrical, and one study was outside the 95% confidence interval, suggesting possible publication bias in the included studies (Figure 2). Additionally, publication bias was assessed using Egger’s tests (Table 2), which revealed no significant indications of publication bias.

## 4 Discussion

The ophthalmic artery is the first major branch of the internal carotid artery, and after entering the orbit, the major branches are the central retinal artery, the short posterior ciliary artery, the long posterior ciliary artery, and the supraorbital artery (Sanomura et al., 2020). Therefore, insufficient perfusion of the carotid artery after stenosis can lead to microcirculatory obstruction in the retina and choroid (Ortuño Andériz et al., 2023). If the obstruction is not removed in time, the metabolism of the retina will be altered, and choroidal perfusion will decrease, leading to microstructural changes (Li et al., 2019). Lee et al. (2019) reported that surgical intervention for ICAS can effectively improve retinal and choroidal perfusion, which in turn confirms the effect of ICAS on ocular blood supply.

Retinal ganglion cells are important neurons for retinal signaling and damage to their function or a reduction in their number can directly affect visual signaling (Bergum et al., 2023). The peripapillary retinal nerve fiber layer is composed of ganglion cell axons that converge on the optic nerve head (Chen et al., 2023). A reduction in the thickness of the retinal nerve fiber layer and ganglion cell complex has been shown to be associated with the development of cognitive impairment, stroke and other brain disorders (Wang et al., 2014). CAS is an independent risk factor for cerebral ischemic disease, and it can lead to cognitive impairment through mechanisms such as chronic hypoperfusion, microembolization and cerebrovascular reactivity impairment (Viticchi et al., 2021). Stevens et al. (2002) reported that carotid artery stenosis can also cause retinal ganglion cell loss and degenerative changes in the optic nerve. In this study, the retinal nerve fiber layer thickness and ganglion cell complex thickness in the eyes on the side of the ICAS group were lower than those in the normal control group, suggesting that retinal nerve tissue is damaged in patients with ICAS. This may be related to the chronic ischemia and hypoxia of the retinal and choroid caused by insufficient ocular perfusion due to persistent ICAS, which causes damage to ganglion cells and nerve fiber layers through oxygen radical damage and impaired axial plasma flow transport in ganglion cells (Lv et al., 2023). In addition, Biberoğlu et al. (2017) reported that the retinal nerve fiber layer thickness in ICAS patients did not change significantly after carotid artery revascularization compared with that in the preoperative period, suggesting that the damage to the optic nerve caused by chronic ischemia may be irreversible.

Choroidal thickness is the distance between the retinal pigment cell layer and the scleral junction of the choroid; it is influenced by choroidal blood flow and vascular filling, and it is an important parameter for assessing choroidal perfusion (Akçay et al., 2016). The choroid is primarily responsible for the supplying of blood and nutrients to the outer retina and is the only blood supply system to the central fovea. Ocular hemodynamic changes caused by carotid artery stenosis can also cause damage to the choroid (Ortuño Andériz et al., 2023). Carotid artery stenosis can lead to reduced choroidal blood flow, and prolonged choroidal hypoperfusion can result in choroidal vascular thinning, capillary occlusion, and even choroidal infarction, ultimately leading to choroidal atrophy and thinning (Seidel et al., 2017). However, changes in the thickness of the choroid in patients with ICAS are controversial. Rabina et al. (2018) reported no significant difference in choroidal thickness between the eyes on the stenotic side and the contralateral eye. Akçay et al. (2016) suggested that this may be related to the body’s compensation to prevent the reduced retinal and choroidal perfusion caused by ICAS, which induces the dilation of the choroidal capillaries on that side of the eye, causing an increase in choroidal thickness. Some studies (Incekalan et al., 2022; Liu X. et al., 2022; Wan et al., 2022) have shown that the choroidal thickness of the eye on the stenotic side is thinner in patients with carotid stenosis than in healthy controls. However, Li et al. (2019) did not observe of altered retinal or choroidal thickness in patients with ICAS. This suggests that there is not a parallel relationship between the degree of ICAS and retinal and choroidal thickness, but it may also be related to the presence of collateral anastomoses between branches of the ophthalmic artery and branches of the external carotid artery (Liu J. et al., 2022). Poor collateral circulation between the internal and external carotid arteries, between the two internal carotid arteries, or even retrograde flow of blood from the ophthalmic artery may result in inadequate blood supply to the eye and changes in the morphological structure of the retina (Lauria et al., 2020).

Numerous studies on the correlation between the ocular microvascular system and systemic diseases have shown that retinal vascular changes can indicate the onset and progression of intracranial and systemic vasculopathies, neurodegenerative diseases, and microvascular disorders (Arrigo et al., 2020). Although the use of OCTA in chronic ocular ischemic diseases caused by carotid artery stenosis is limited, OCTA has great potential for observing the retinal capillary network, especially the deep vascular network, with advantages not available with traditional ophthalmic ancillary examination techniques such as fundus angiography. Animal studies (Wang et al., 2016) have shown that retinal vascular density is reduced in rats with bilateral common carotid artery ligation, and a study by Lahme et al. (2018) in 25 patients with severe unilateral ICAS revealed that the overall vessel density of the radial peripapillary capillaries and the superficial capillary plexus was reduced compared to that of healthy controls. In this study, the overall vessel density of the radial peripapillary capillary density was lower in ICAS patients than in healthy controls, suggesting that microcirculatory disturbances may occur in the retina of patients with ICAS.

Limitations of this study: (a) Data such as BCVA and IOP were not statistically analyzed due to differences in the way the data were analyzed in the included literature; (b) Further comparisons of retinal and choroidal changes for different degrees of ICAS could not be made due to the limited number of included studies; (c) In addition, differences in the examination equipment used in the included studies, the duration of illness and complications of the included patients also make the results subject to a certain degree of error, and these factors need to be further considered in the studies for follow-up; (d) The observation of the microstructure of the retina and choroid is not precise enough, and further detailed analysis using relevant software is needed; (e) There is significant heterogeneity among various studies, so we conducted subgroup analysis and sensitivity analysis, but did not find the exact source of heterogeneity, which may be related to the limited number of samples included.

## 5 Conclusion

The meta-analysis in this study revealed that carotid artery stenosis affects the morphology and structure of the retina and choroid in patients with ICAS. Chronic ocular ischemic disease due to carotid artery stenosis has an insidious onset and varied ocular manifestations, which may lead to severe vision loss, blindness, or even life-threatening cerebrovascular accidents if not treated in time. However, it is often difficult to establish a clinical link between ocular symptoms and carotid artery stenosis, which makes it easy to miss or misdiagnose the disease and requires the attention of ophthalmologists. Before patients with internal carotid artery stenosis show typical ocular ischemia, the thickness of the retina and choroid as well as the blood flow density can be quantitatively analyzed by OCT and OCTA in these patients, so that the impact of internal carotid artery stenosis on the ocular blood supply can be detected earlier.

## Data availability statement

The datasets presented in this study can be found in online repositories. The names of the repository/repositories and accession number(s) can be found in the article/Supplementary material.

## Author contributions

XH: Conceptualization, Methodology, Formal analysis, Writing – original draft. ZL: Writing – review and editing, Methodology, Supervision. XB: Writing – review and editing, Methodology, Supervision. YD: Writing – review and editing, Visualization, Supervision. YL: Writing – review and editing, Visualization, Investigation. JW: Writing – review and editing, Visualization, Investigation. WZ: Writing – review and editing, Supervision. CJ: Conceptualization, Funding acquisition, Project administration, Writing – review and editing.
