# Supplementary material for: Changes in the retina and choroid in patients with internal carotid artery stenosis: a systematic review and meta-analysis
Source: Front Neurosci. 2024 Apr 15;18:1368957. doi: 10.3389/fnins.2024.1368957 (PMC11056587; doi:10.3389/fnins.2024.1368957)
Supplement: Supplementary file 1 [file Data_Sheet_1.doc]

## Supplementary Table S1: The search strategy

## The search strategy of Pubmed

| **No.** | **Search items** |
| --- | --- |
| #1 | "Internal carotid artery stenosis"[Mesh] |
| #2 | (((((((((((((((((((((((((((((((Carotid Stenoses[Title/Abstract]) OR (Stenoses, Carotid[Title/Abstract])) OR (Stenosis, Carotid[Title/Abstract])) OR (Carotid Artery Narrowing[Title/Abstract])) OR (Artery Narrowing, Carotid[Title/Abstract])) OR (Artery Narrowings, Carotid[Title/Abstract])) OR (Headache, MigraineCarotid Artery Narrowings[Title/Abstract])) OR (Narrowing, Carotid Artery[Title/Abstract])) OR (Narrowings, Carotid Artery[Title/Abstract])) OR (Carotid Artery Stenosis[Title/Abstract])) OR (Artery Stenoses, Carotid[Title/Abstract])) OR (Artery Stenosis, Carotid[Title/Abstract])) OR (Carotid Artery Stenoses[Title/Abstract])) OR (Stenoses, Carotid Artery[Title/Abstract])) OR (Stenosis, Carotid Artery[Title/Abstract])) OR (Internal Carotid Artery Stenosis[Title/Abstract])) OR (Common Carotid Artery Stenosis[Title/Abstract])) OR (Stenosis, Common Carotid Artery[Title/Abstract])) OR (External Carotid Artery Stenosis[Title/Abstract])) OR (Stenosis, External Carotid Artery[Title/Abstract])) OR (Carotid Artery Plaque[Title/Abstract])) OR (Artery Plaque, Carotid[Title/Abstract])) OR (Artery Plaques, Carotid[Title/Abstract])) OR (Carotid Artery Plaques[Title/Abstract])) OR (Plaque, Carotid Artery[Title/Abstract])) OR (Plaques, Carotid Artery[Title/Abstract])) OR (Carotid Ulcer[Title/Abstract])) OR (Carotid Ulcers[Title/Abstract])) OR (Ulcer, Carotid[Title/Abstract])) OR (Ulcers, Carotid[Title/Abstract])) OR (Ulcerating Plaque, Carotid Artery[Title/Abstract])) OR (Plaque, Ulcerating, Carotid Artery[Title/Abstract])) OR (Carotid Artery Ulcerating Plaque[Title/Abstract]) |
| #3 | #1 OR #2 |
| #4 | (((((Optical coherence tomography [Title/Abstract]) OR (OCT[Title/Abstract])) OR (optical coherence tomography angiography [Title/Abstract])) OR (OCT angiography [Title/Abstract])) OR (angio-OCT[Title/Abstract])) OR (OCTA[Title/Abstract]) |
| #5 | #3 AND #4 |

## The search strategy of Embase

| **No.** | **Search items** |
| --- | --- |
| #1 | 'carotid artery stenosis'/exp |
| #2 | 'Carotid Stenoses':ab,ti OR'Stenoses, Carotid':ab,ti OR'Stenosis, Carotid':ab,ti OR'Carotid Artery Narrowing':ab,ti OR'Artery Narrowing, Carotid':ab,ti OR'Artery Narrowings, Carotid':ab,ti OR' Headache, MigraineCarotid Artery Narrowings':ab,ti OR' Narrowing, Carotid Artery':ab,ti OR' Narrowings, Carotid Artery':ab,ti OR' Carotid Artery Stenosis':ab,ti OR' Artery Stenoses, Carotid':ab,ti OR' Artery Stenosis, Carotid':ab,ti OR' Carotid Artery Stenoses':ab,ti OR' Stenoses, Carotid Artery':ab,ti OR' Stenosis, Carotid Artery':ab,ti OR' Internal Carotid Artery Stenosis':ab,ti OR' Common Carotid Artery Stenosis':ab,ti OR' Stenosis, Common Carotid Artery':ab,ti OR' External Carotid Artery Stenosis':ab,ti OR' Stenosis, External Carotid Artery':ab,ti OR' Carotid Artery Plaque':ab,ti OR' Artery Plaque, Carotid':ab,ti OR' Artery Plaques, Carotid':ab,ti OR' Carotid Artery Plaques':ab,ti OR' Plaque, Carotid Artery':ab,ti OR' Plaques, Carotid Artery':ab,ti OR' Carotid Ulcer':ab,ti OR' Carotid Ulcers':ab,ti OR' Ulcer, Carotid':ab,ti OR' Ulcers, Carotid':ab,ti OR' Ulcerating Plaque, Carotid Artery':ab,ti OR' Plaque, Ulcerating, Carotid Artery':ab,ti OR' Carotid Artery Ulcerating Plaque |
| #3 | #1 OR #2 |
| #4 | 'optical coherence tomography': ab,ti OR 'oct':ab,ti OR 'optical coherence tomography angiography':ab,ti OR 'oct angiography':ab,ti OR 'angio-oct':ab,ti OR 'octa':ab,ti |
| #5 | #3 AND #4 |

**Supplementary Table S2:** The quality assessment of the literature

| References | Selection | Comparability | Outcomes | Score |
| --- | --- | --- | --- | --- |
| Sayin et al., 2015 | *** | ** | ** | 7 |
| Heßler et al., 2015 | *** | * | *** | 7 |
| Çakır et al., 2017 | **** | * | *** | 8 |
| Biberoğlu et al., 2017 | *** | ** | ** | 7 |
| Wang et al., 2017 | **** | * | *** | 8 |
| Lahme et al., 2018 | **** | * | *** | 8 |
| Li et al., 2019 | **** | * | *** | 8 |
| Biberoglu et al., 2020 | **** | ** | ** | 8 |
| Pierro et al., 2021 | *** | ** | *** | 8 |
| Dagdelen and Muz, 2021 | *** | *** | ** | 8 |
| Liu X. et al., 2022 | *** | * | *** | 7 |
| Wan et al., 2022 | *** | ** | ** | 7 |
| Incekalan et al., 2022 | *** | ** | ** | 7 |

**Supplementary Figure S1: The detailed forest plots (ICAS vs. HC)**

**
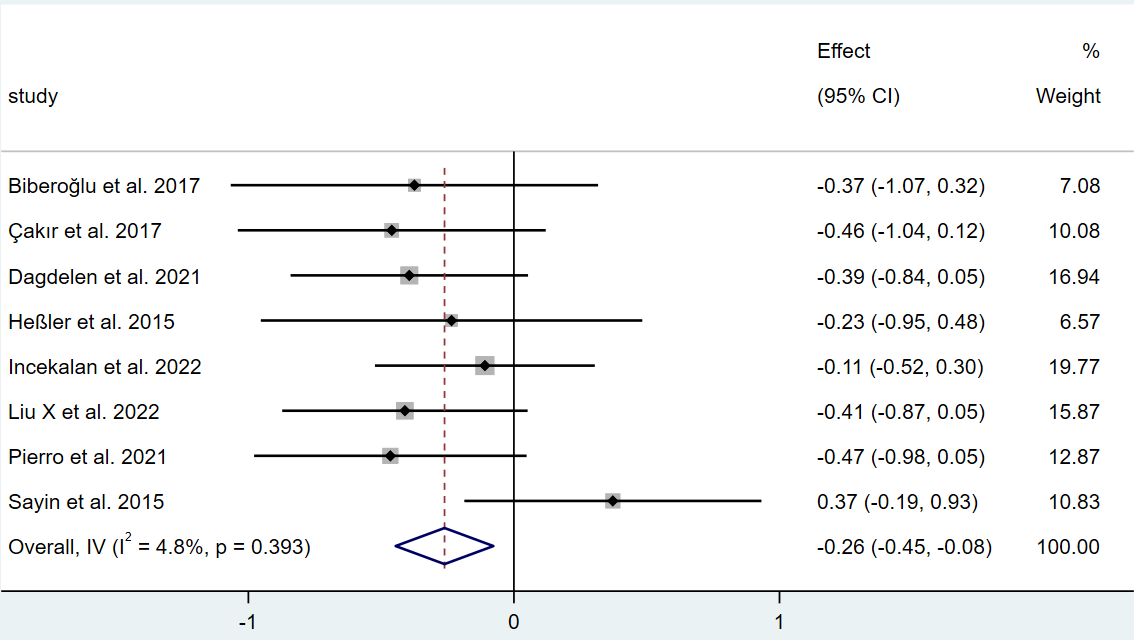
**A**
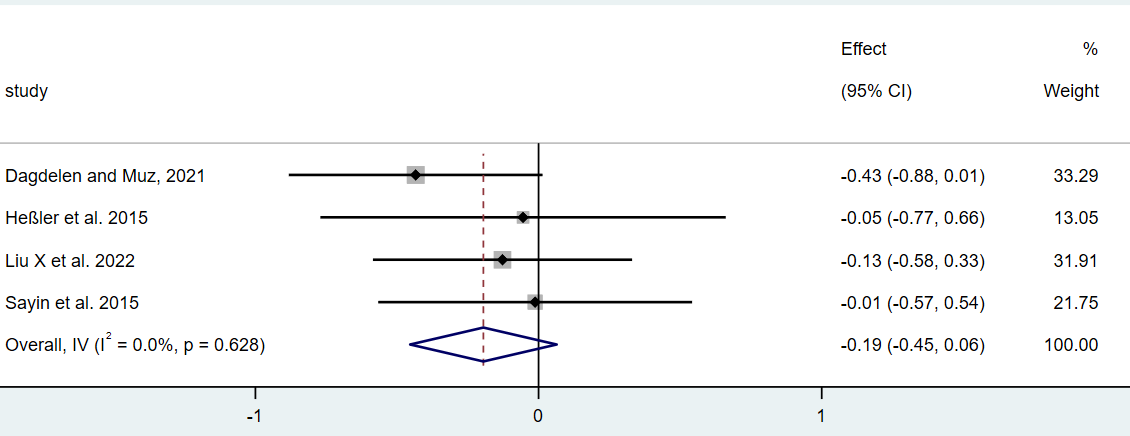
**B

**
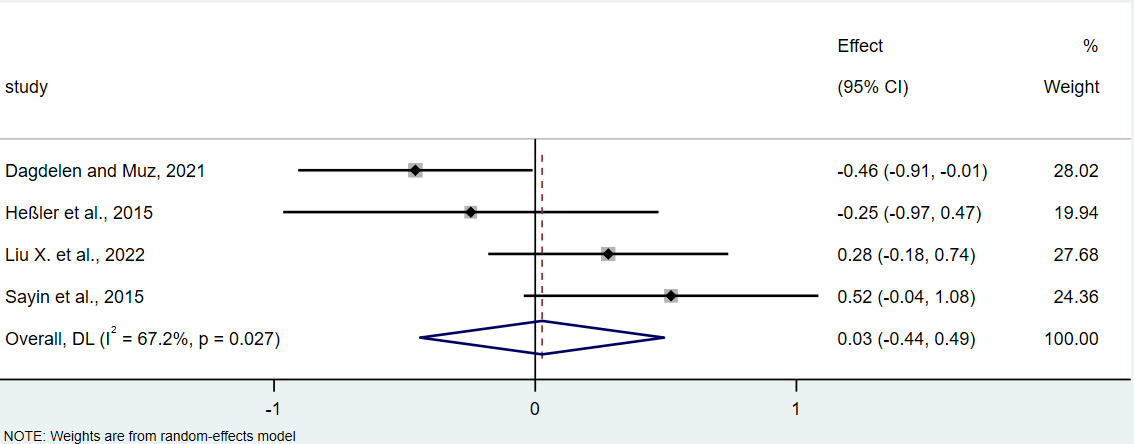
**C**
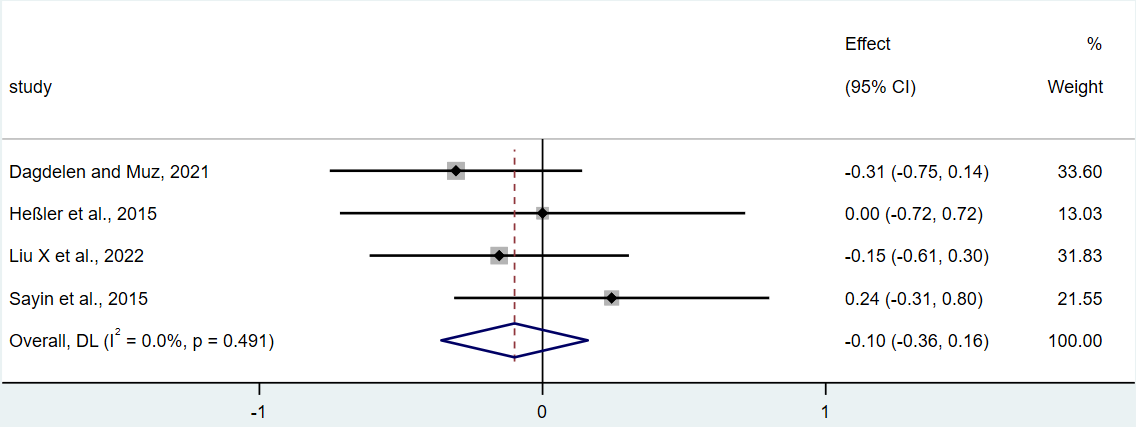
**D

**
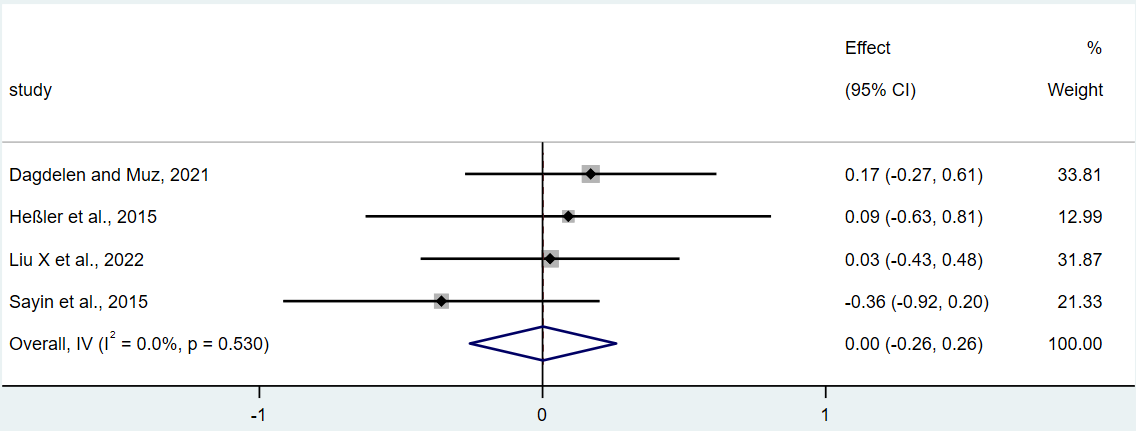
**E**
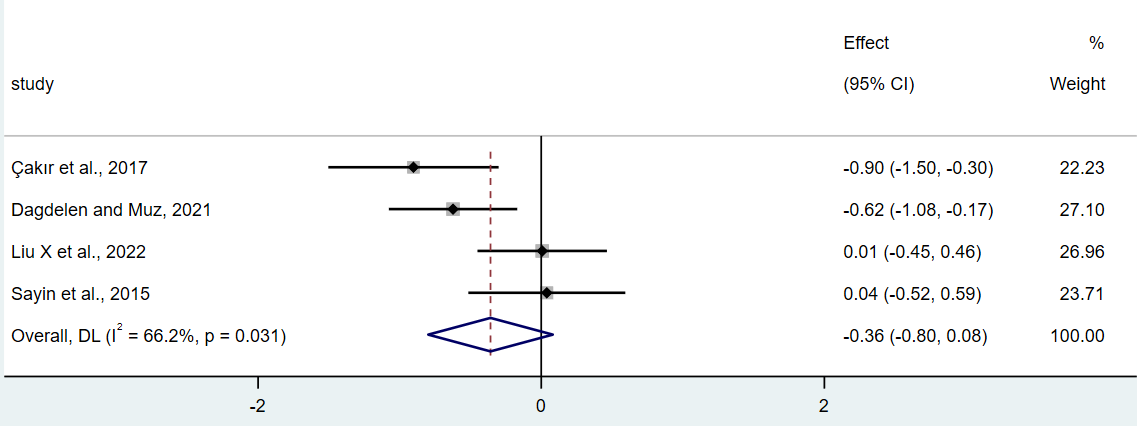
**F

**
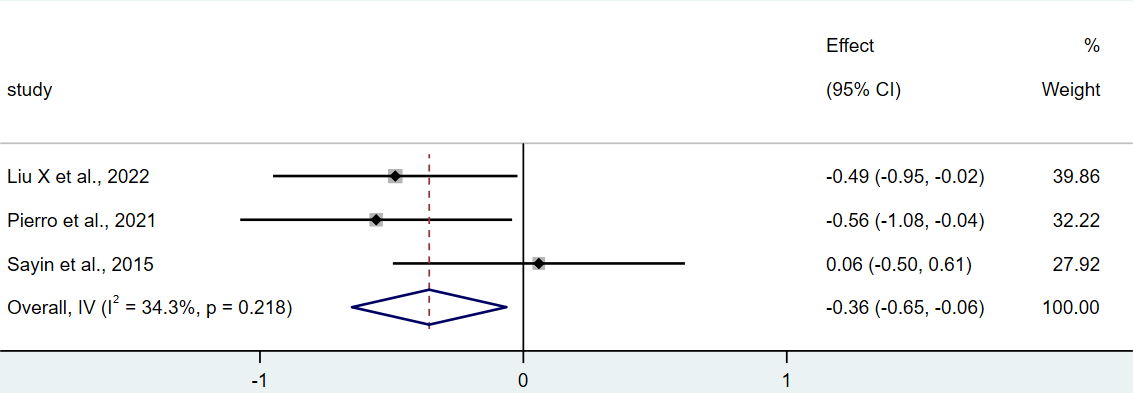
**G**
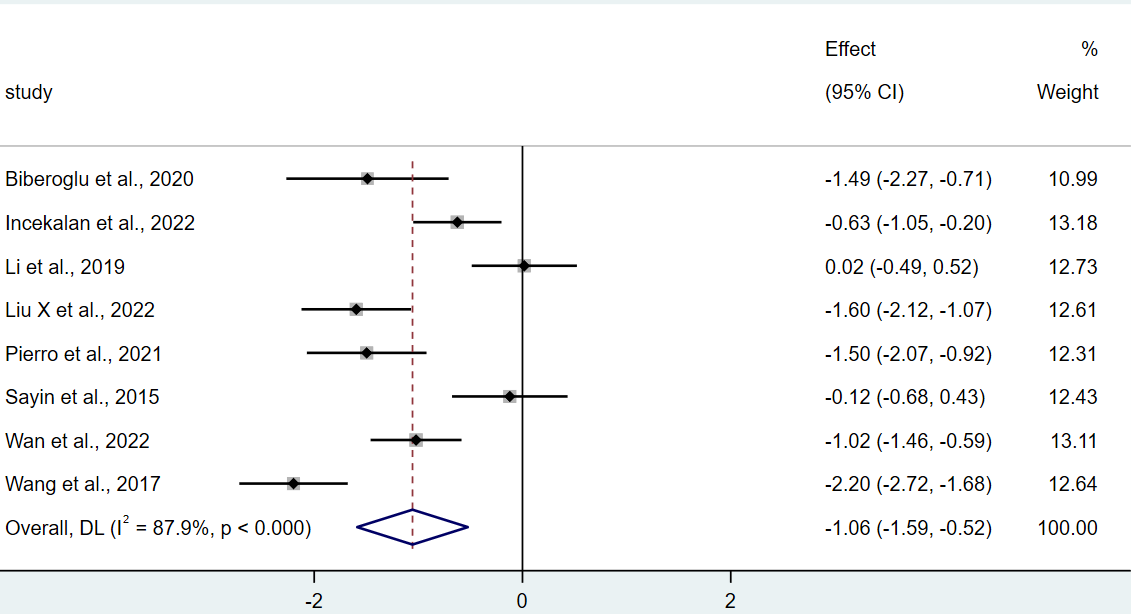
**H


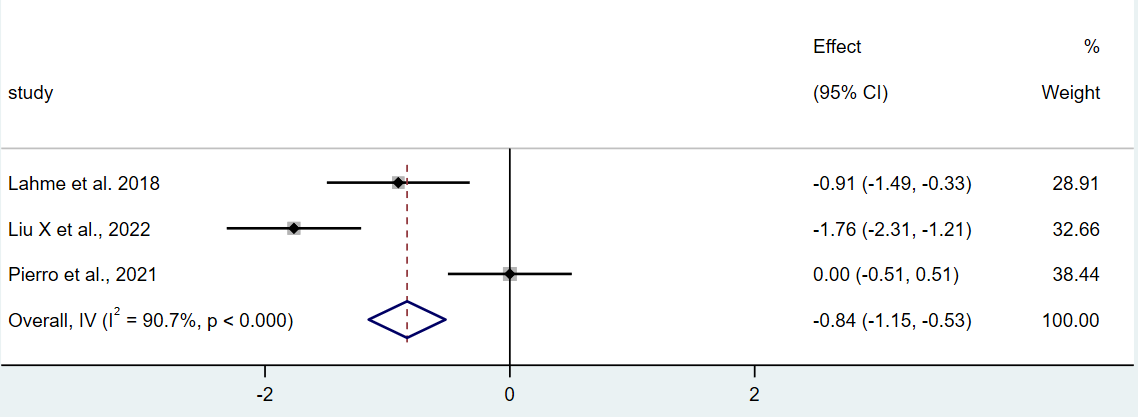
I
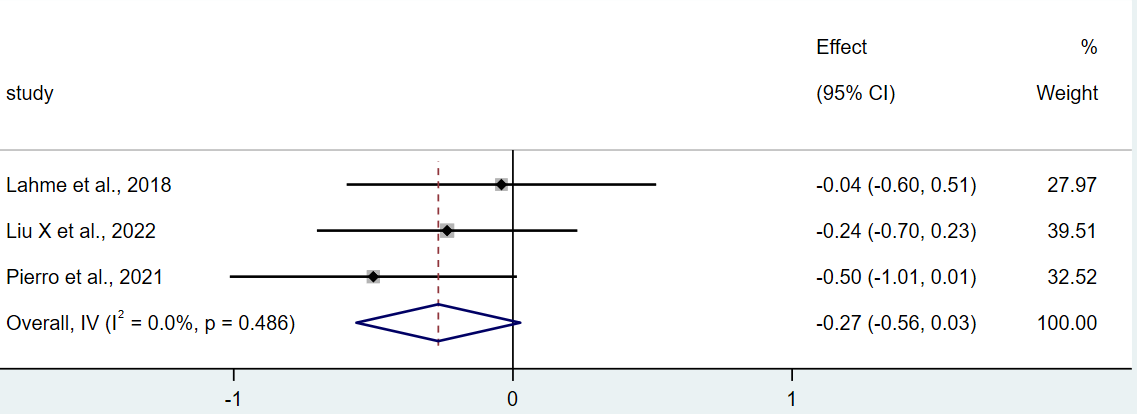
J


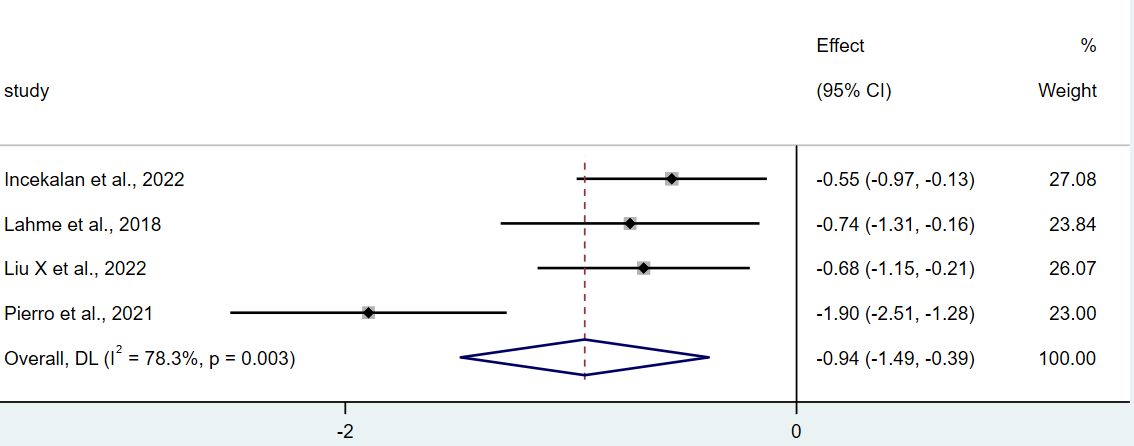
K
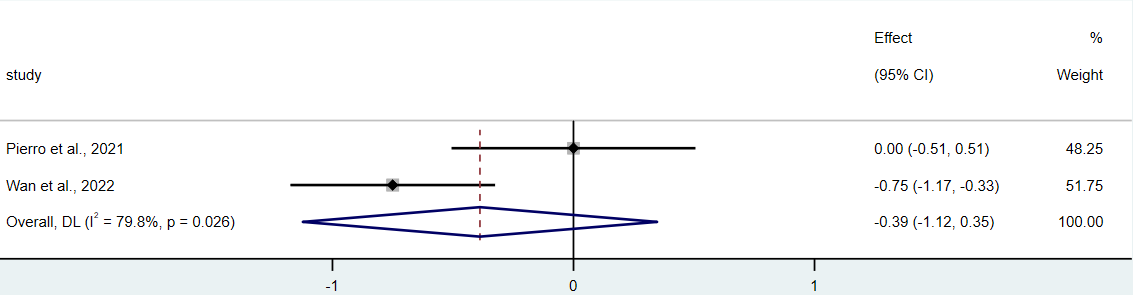
L

A: Forest plots of the average thickness of the pRNFL;B: Forest plots of the thickness of the pRNFL in the inferior; C: Forest plots of the thickness of the pRNFL in then nasal; D: Forest plots of the thickness of the pRNFL in the superior; E: Forest plots of the thickness of the pRNFL in the temporal; F: Forest plots of the thickness of macular thickness; G: Forest plots of the thickness of the thickness of the ganglion cell complex; H: Forest plots of the thickness of choroid; I: Forest plots of the vessel density of superficial capillary plexus; J: Forest plots of the vessel density of deep capillary plexus; K: Forest plots of the vessel density of radial peripapillary capillaries plexus; L: Forest plots of the vessel density of choriocapillaris.

**Supplementary Figure S2: Sensitivity Analysis Results of the Meta-analysis**


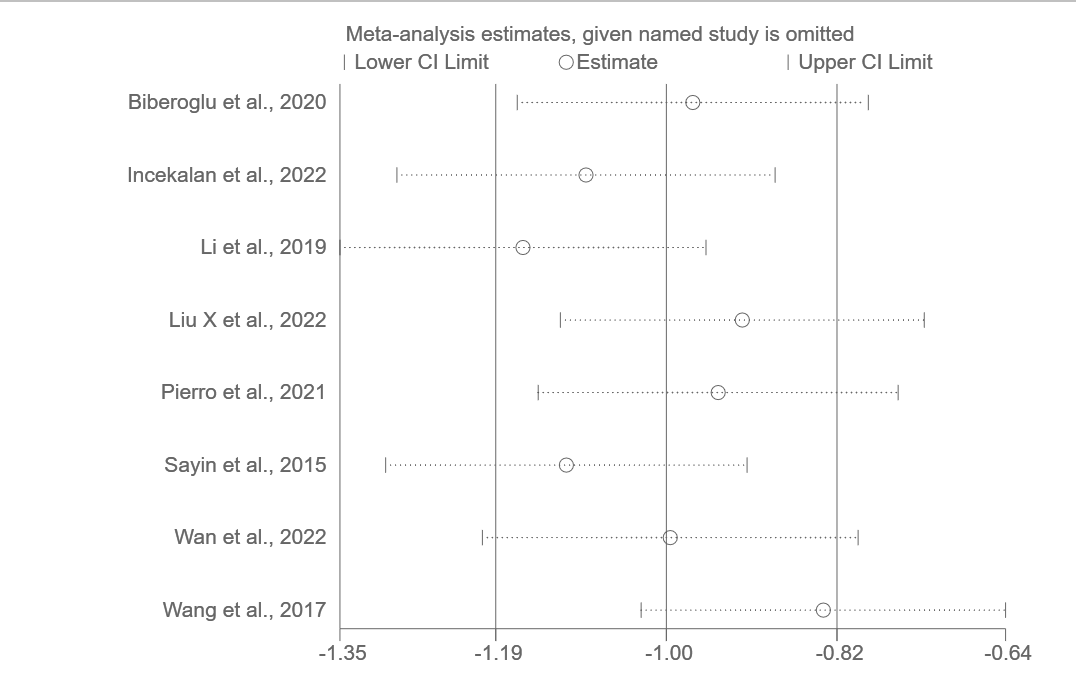
A


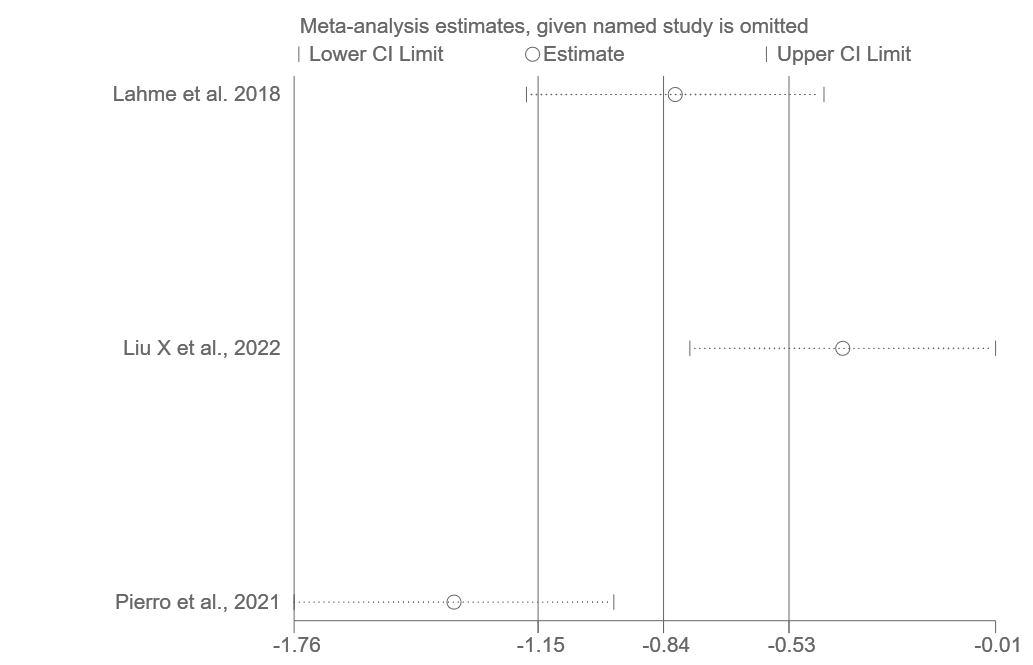
 B


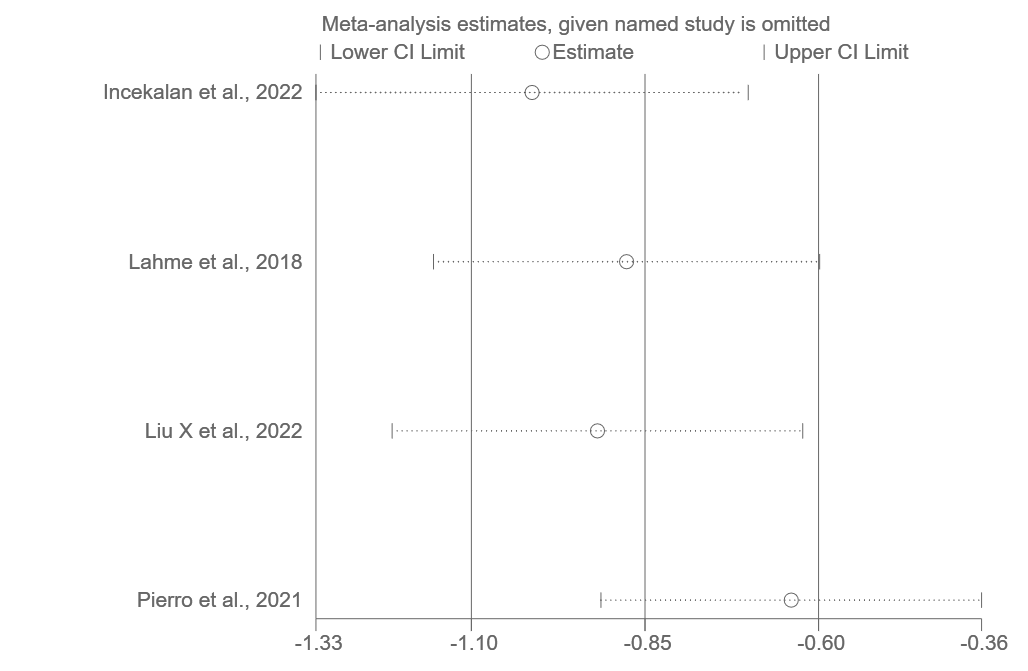
 C

A: the Sensitivity analysis of the Choroidal thickness CT; B: the Sensitivity analysis of the vessel density of superficial capillary plexus; C: the sensitivity analysis of the vessel density of radial peripapillary capillaries plexus

**Supplementary Table S3: Subgroup Analysis Results of the Meta-ana**lysis

| **Percentage of stenosis, %** | **No. Of studies** | **Heterogeneity** | |  | **Overall effect** | |
| --- | --- | --- | --- | --- | --- | --- |
|  |  | ***I^2^*(%)** | ***P*** |  | ***Z*** | ***P*** |
| **CT** |  |  |  |  |  |  |
| ≥70 | 2 | 87.3 | 0.005 |  | -1.137 | 0.255 |
| ≥50 | 6 | 89.1 | 0.000 |  | -3.680 | 0.000 |
| **pRNFL-Nasal** |  |  |  |  |  |  |
| ≥70 | 1 | - | - |  | 1.807 | 0.071 |
| ≥50 | 3 | 61.6 | 0.074 |  | -0.523 | 0.601 |
| **CMT** |  |  |  |  |  |  |
| ≥70 | 1 | 69.5 | 0.038 |  | -1.813 | 0.070 |
| ≥50 | 3 | - | - |  | 0.139 | 0.889 |
| **Whole-image-SCP** |  |  |  |  |  |  |
| ≥70 | 1 | - | - |  | -3.061 | 0.002 |
| ≥50 | 2 | 95.3 | 0.000 |  | -0.996 | 0.319 |
| **Whole-image-RPC** |  |  |  |  |  |  |
| ≥70 | 1 | - | - |  | -2.521 | 0.012 |
| ≥50 | 2 | 85.3 | 0.001 |  | -2.660 | 0.008 |

|  |
| --- |

| **TypeofOCT** | **No.Ofstudies** | **Heterogeneity** | |  | **Overalleffect** | |
| --- | --- | --- | --- | --- | --- | --- |
|  |  | ***I^2^*(%)** | ***P*** |  | ***Z*** | ***P*** |
| **CT** |  |  |  |  |  |  |
| SD-OCT | 3 | 72.8 | 0.025 |  | -3.540 | 0.000 |
| EDI-OCT | 4 | 94.1 | 0.000 |  | -1.771 | 0.077 |
| SS-OCT | 1 | - | - |  | -3.891 | 0.000 |
| **pRNFL-Nasal** |  |  |  |  |  |  |
| SD-OCT | 3 | 72.4 | 0.027 |  | -0.212 | 0.832 |
| EDI-OCT | 1 | - | - |  | 1.192 | 0.233 |
| **CMT** |  |  |  |  |  |  |
| SD-OCT | 3 | 64.5 | 0.060 |  | -1.867 | 0.062 |
| EDI-OCT | 1 | - | - |  | 0.029 | 0.977 |
